# SHP2 inhibition improves celastrol-induced growth suppression of colorectal cancer

**DOI:** 10.3389/fphar.2022.929087

**Published:** 2022-09-01

**Authors:** Linxi Zhang, Xuefei Hu, Qingying Meng, Ye Li, Hao Shen, Yating Fu, Fan Zhang, Jiahui Chen, Wei Zhang, Wenjun Chang, Yamin Pan

**Affiliations:** ^1^ Department of Navy Environmental and Occupational Health, Faculty of Naval Medicine, Navy Military Medical University, Shanghai, China; ^2^ Department of Digestive Endoscopy, Shuguang Hospital, Shanghai University of Traditional Chinese Medicine, Shanghai, China; ^3^ Department of Colorectal Surgery, Changhai Hospital, Navy Military Medical University, Shanghai, China

**Keywords:** colorectal cancer, celastrol, SHP2, combined chemotherapy, growth suppression, AKT/ERK pathway

## Abstract

This study aimed to explore novel targets for celastrol sensitization in colorectal cancer (CRC) based on differentially regulated signals in response to high- or low-dose celastrol. Targeting signals were investigated using Western blotting or phosphorylated receptor tyrosine kinase (RTK) arrays. Corresponding inhibitors for the signals were individually combined with low-dose celastrol for the assessment of combined anti-CRC effects, based on proliferation, apoptosis, colony assays, and xenograft models. The potential mechanism for the combination of celastrol and SHP2 inhibition was further examined. Low-dose celastrol (<1 µM) did not effectively suppress AKT and ERK signals in CRC cells compared to high-dose celastrol (>1 µM). However, when combined with an AKT or ERK inhibitor, low-dose celastrol could cooperatively suppress CRC proliferation. Furthermore, failed AKT or ERK inhibition by low-dose celastrol may be due to reactivated RTK-SHP2 signaling with negative feedback. The combination of celastrol and the SHP2 inhibitor resulted in greatly reduced AKT and ERK signals, as well as greater inhibition of CRC growth than celastrol alone. Moreover, the mechanism underlying combination suppression was also involved in the activation of immune cell infiltration (mainly for CD8^+^ cells) in CRC tissues. Failure to inhibit RTK-SHP2-AKT/ERK signaling contributed to the lack of CRC growth suppression by low-dose celastrol. However, the combination of celastrol and the SHP2 inhibitor resulted in synergistic inhibition of CRC growth and provided a promising therapeutic target.

## Introduction

Colorectal cancer (CRC) is the third most prevalent cancer worldwide, with 1.9 million patients newly diagnosed in 2020 (Bertotti et al., 2015). CRC is a heterogeneous disease of the intestinal epithelium defined by various activating mutations in receptor tyrosine kinases (RTKs), as well as gain- or loss-of-function mutations in downstream components of RTK-activated intracellular pathways, some of which can occur simultaneously in the same tumor (Bertotti et al., 2015; Janney et al., 2020), and is also characterized by the dysregulated immune response. Despite the development of advanced cancer treatments, the 5-year survival rate is still poor, especially for metastatic CRC. Fewer than 20% of patients survive beyond 5 years. The primary reason for treatment failure is a marked resistance of the cancer to chemotherapy. In patients with metastatic CRC, the occurrence of drug resistance is more than 90% (Longley and Johnston, 2005). Therefore, novel treatment strategies are needed to overcome or evade drug resistance.

Natural products continue to serve as an important and valuable source of drug discovery (Corson and Crews, 2007). Celastrol (also named tripterine) is a potent chemotherapeutic agent, with multiple molecular targets, and is effective against a variety of cancers, including breast (Cao et al., 2015), liver (Du et al., 2020), lung, ovarian, prostate (Shao et al., 2013), and colorectal cancer (Kannaiyan et al., 2011; Rajendran et al., 2012; Chen et al., 2018; Kashyap et al., 2018; Ng et al., 2019; Nouri et al., 2019). However, a concern regarding the clinical translation of celastrol is its narrow therapeutic window regarding the dose and the undesired side effects. The therapeutic dose of celastrol for various tumor xenograft models is in the range of 3–5 mg/kg (Yang et al., 2006), which may induce systemic toxicities, including infertility toxicity, cardiotoxicity (Liu et al., 2019), hepatotoxicity (Jin et al., 2019), hematopoietic system toxicity, and nephrotoxicity (Wu et al., 2018). However, lower celastrol doses, though safe, show limited antitumor efficacy. Other drawbacks of celastrol include low bioavailability and poor water stability. Therefore, further research is required to understand and overcome these limits (Guo et al., 2017; Yu et al., 2018; Shi et al., 2020; Wagh et al., 2021).

Lowering the dosage of celastrol by combining with other agents may effectively reduce its related adverse effects, improve its therapeutic effect, and prevent multidrug resistance. Thus, exploring combination therapy may offer more opportunities for the clinical translation of celastrol. Celastrol exerts its anticancer effects by modulating diverse signal transduction pathways and oncogenic molecular targets (Yang et al., 2006). Although the combination of celastrol with multiple inhibitors targeting RTKs (Raja et al., 2011), apoptosis proteins, HSP90, NF-κB (Zheng et al., 2014), and DNA damage and repair molecules have been reported to play significant additive and synergistic roles in preclinical cancer models, combination therapy in CRC is still poorly understood (Zhu et al., 2012; Kim et al., 2017; Shanmugam et al., 2018; Gao et al., 2019; Metselaar et al., 2019).

In this study, we first investigated the inhibitory effects of low-dose celastrol in CRC. Then, we carried out the combined therapeutic employment of celastrol and AKT/ERK inhibitors to discuss the underlying mechanism of the limited suppressive function of low-dose celastrol. Moreover, SHP2, a key mediator of multiple RTK and AKT/ERK signaling, served as a target for CRC. We further explored the synergistic function and molecular mechanism of low-dose celastrol and the SHP2 inhibitor regardless of the KRAS or BRAF mutations in CRC. Finally, we observed the anti-CRC function of low-dose celastrol and SHP2 inhibition in xenograft animal models *in vivo.* Taken together, our findings highlighted that celastrol administration with SHP2 inhibition may present a promising molecular therapeutic strategy for CRC.

## Materials and methods

### Cells and reagents

Cell lines were obtained from the American Type Culture Collection (ATCC), which routinely performs cell line authentication testing using short tandem repeat analysis, and maintained in Dulbecco’s modified Eagle’s medium (DMEM; for SW480 and Caco2 cells) or RPMI-1640 (SW620, RKO, and MC38 cells) supplemented with 10% heat-inactivated fetal bovine serum (10099141, Gibco, Carlsbad, CA, United States), 100 Uml^−1^ penicillin, and 100 mg/ml streptomycin (150700063, Gibco, Carlsbad, CA, United States) at 37°C in a humidified atmosphere containing 5% carbon dioxide. Celastrol (HY-13067), SHP2 inhibitor (SHP099, HY-100388), EGFR inhibitor (BPI-2009H, HY-15164), HGFR inhibitor (PHA-665752, HY-11107), AXL inhibitor (SGI-7079, HY12964), EPHA2 inhibitor (ALW-II-41–27, HY-18007), and ALK inhibitor (GSK1838705A, HY-13020) were purchased from MedChemExpress, Monmouth, United States. The AKT-1/2/3 inhibitor (MK-2206, S1078) and ERK-1/2 inhibitor (GDC0994, S7554) were purchased from Selleck, Shanghai, China.

### Cell proliferation assays

CRC cells were seeded in triplicate in 96-well plates at 3,000–5,000 cells per well and were exposed to SHP099 alone, MK-2206 alone, GDC0994 alone, or their combinations with indicated concentrations. The number of viable cells was assessed at 48 h using the cell counting kit-8 (Dojindo, Shanghai, China) according to the manufacturer’s instructions. The absorbance at 450 nm was measured to reflect the viable cell population.

### Real-time apoptosis assays

Polarity Sensitive Indicator of Viability & Apoptosis (*p*SIVA) assays were employed to monitor the dynamic apoptosis of CRC cells induced by celastrol and the indicated inhibitors, using *p*SIVA™-IANBD Apoptosis/Viability Kits (NBP2-29382, Novus Biologicals, Centennial, United States) according to the manufacturer’s instructions. Briefly, celastrol and the indicated inhibitors were added into the medium of CRC cells cultured in 96-well plates alone or in combination to induce apoptosis. Thereafter, the *p*SIVA-IANBD probe (1.5 μL) was added directly to the culture medium of each well. We then monitored the cells under physiological conditions (37°C, 5% CO_2_) using time-lapse microscopy. A × 40 phase objective was used to collect transmission images, while *p*SIVA was detected in the FITC fluorescent channel. Images were taken every 2–4 h at each field of view.

### Colony formation assays

For colony formation assays, the cells were first cultured in six-well plates (Corning, NY, United States) at a density of 1.0 × 10^4^ cells/well, and the regular medium supplemented with the inhibitors was refreshed every 2 days. After culturing for 1–2 weeks, the resulting colonies were fixed with 4% formaldehyde and stained with crystal violet solution for counting. The assay was performed in triplicate. The plates were scanned using a photo-scanner, and the cell growth was quantified using ImageJ software.

### Animal studies

Animal experiments were carried out according to the Institutional Animal Care and institutional guidelines for the proper and humane use of animals in research. Four-week-old nu/nu athymic male BALB/c mice and eight-week-old male C57BL/6 mice were obtained from Shanghai Jihui Experimental Animal Breeding Company (Shanghai, China) and maintained in pressurized ventilated caging. Subcutaneous tumors were generated by transplanting 0.5–1.0 × 10^7^ tumor cells (SW620 and RKO for BALB/c mice and MC38 for C57BL/6 mice) in phosphate-buffered saline (PBS) into the right flank (100 μl/mouse) and randomized approximately 10–12 days postimplantation (size >100 mm^3^). Mice were treated with SHP099 alone (40 mg/kg), celastrol alone (1 mg/kg), or their combination with the indicated doses. SHP099 was formulated in 30% hydroxypropyl-β-cyclodextrin, and celastrol was dissolved in dimethyl sulfoxide (DMSO) water and administered *via* oral gavage. All inhibitors were administered orally each day. Tumor dimensions were measured using Vernier calipers at an interval of 3 days, and tumor volumes were calculated using the following formula: π/6 × larger diameter × (smaller diameter)^2^. Animals were sacrificed *via* CO_2_ euthanasia when tumors reached the maximum allowed size or when signs of ulceration were evident. After image analysis, tumor specimens were isolated and further processed for Western blotting and immunohistochemistry (IHC) examination.

### Western blotting

Cells were washed with PBS once and incubated on ice for 30 min using RIPA lysis buffer and extraction buffer (89900, Thermo Fisher, CA, United States) with added Pierce Protease and Phosphatase Inhibitor Mini Tablets (A32959, Thermo Fisher, CA, United States). The suspension was then centrifuged for 15 min (14,000 ×*g*) at 4°C. The protein concentration was determined using bicinchoninic acid (BCA) reagent (Dingguo, Beijing, China). Equal amounts of protein (10–50 μg) were separated via sodium dodecyl sulfate-polyacrylamide gel electrophoresis, transferred to polyvinylidene difluoride membranes (Millipore), immunoblotted with specific primary and secondary antibodies, and detected by chemiluminescence using ECL detection reagents from Millipore. Antibodies for Western blotting against *p*-ERK1/2 (T202/Y204, 1:2000, 4,370), ERK1/2 (1:1,000, 4,695), AKT (1:1,000, 4,691), and *p*-AKT (S473, 1:1,000, 4,060) were purchased from Cell Signaling Technology. Antibodies against GAPDH (1:5,000, ab181602) and *p*-SHP2 (Y542, 1:1,000, ab62322) were purchased from Abcam.

### Receptor tyrosine kinase arrays

Human phospho-RTK arrays (R&D Systems) were utilized to examine the RTK levels in response to low-dose celastrol exposure, according to the manufacturer’s instructions. Briefly, cells were washed with cold PBS and lysed in NP40 lysis buffer. Thereafter, lysates (300 μg) were incubated with blocked membranes overnight. Membranes were subsequently washed, exposed to chemiluminescent reagents, and exposed to the X-ray film. Quantification of pixels was performed by employing densitometry analysis using Adobe CS2 and Fuji Film Multi-Gauge software.

### Immunohistochemistry

Primary rabbit poly-antibodies to mouse CD8 (1:500, GB11086, Servicebio, Wuhan, China), mouse CD4 (1:200, GB13064-2, Servicebio, Wuhan, China), and mouse GZMB (1:200, 46890s, CST, MA, United States) were used for immunostaining of formalin-fixed, paraffin-embedded tumor tissue slides prepared using isolated MC38 tumor tissues, which were exposed to SHP099 alone, celastrol alone, or a combination of the two. Briefly, tissue slides were deparaffinized, rehydrated using graded alcohol, and subjected to heat-induced epitope retrieval in citrate-based buffer (pH 6.2) for 20 min. After incubation in a protein block solution for 30 min, the tissue sections were incubated in primary antibodies overnight at 4°C, followed by incubation in the horse-radish peroxidase polymer-conjugated secondary antibody (Manxin, Fuzhou, China). Proteins recognized by antibodies were visualized using DAB (DAB-2031, Manxin, Fuzhou, China). The percentage of CD8^+^, CD4^+^, and GZMB^+^ cells was recorded for analysis.

### Statistics

Drug combination dose–response matrix data were analyzed using the SynergyFinder R package under the R 4.1.1 platform (Ianevski et al., 2017). Quantitative data are presented as the mean ± standard deviation. Two-sided Student’s *t*-tests were used for comparisons of the means of data between two groups, and one-way ANOVA with post hoc Tukey’s test was used for comparisons among multiple independent groups. Statistical tests were performed using SPSS version 22.0 for Windows (SPSS, Chicago, IL, United States), and the statistical significance was set at *p* < 0.05.

## Results

### Low-dose celastrol did not inhibit colorectal cancer due to sustained AKT/ERK activation

**FIGURE 1:**
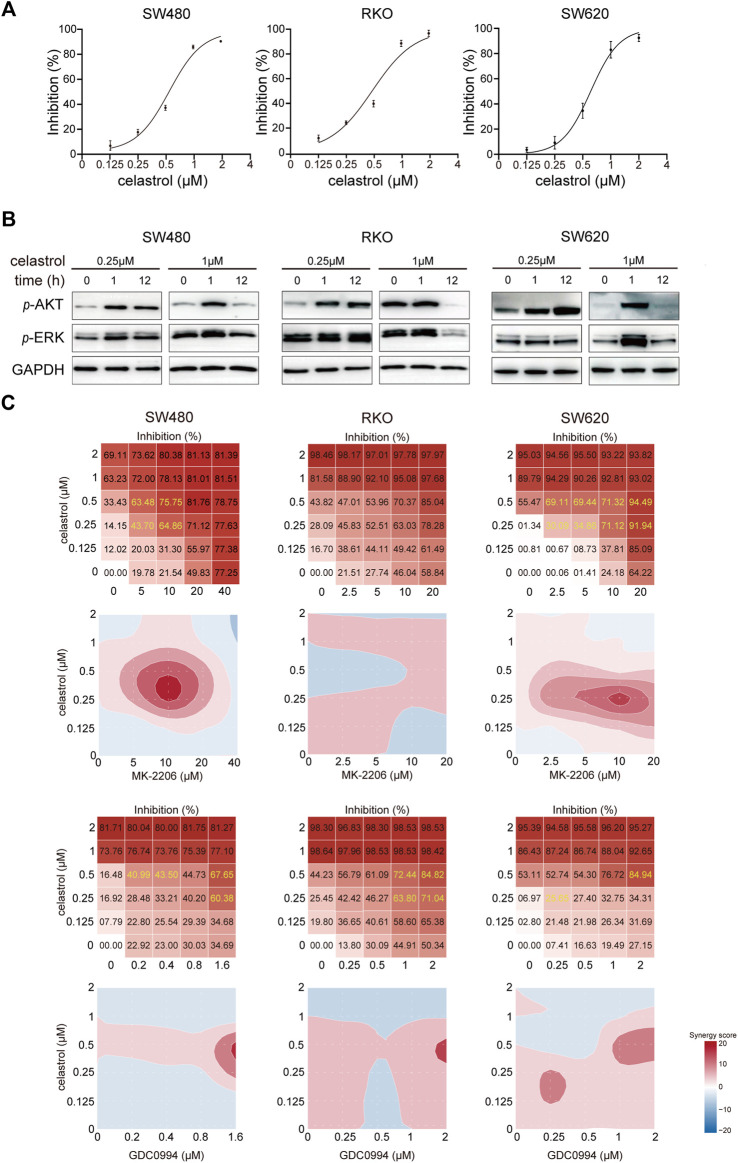
Low-dose celastrol treatment results in sustained AKT/ERK activation in CRC cells. **(A)** Growth inhibition curves of CRC cells in response to celastrol treatment with different doses. **(B)** Expression of *p*-AKT and *p*-ERK in CRC cells received celastrol treatment with 0.25 and 1 µM for 0, 1, and 12 h. **(C)** Inhibitory effect and synergy score of combined treatment of AKT/ERK inhibitor with celastrol in CRC cells.

Growth of CRC cells (SW480, RKO, and SW620) varied in response to different celastrol concentrations (from 0.125 to 2 μM; Figure 1A). Inhibition curves raised sharply when the concentration of celastrol increased from 0.5 to 1.0 µM, as compared with other intervals in all three CRC cell lines (Figure 1A), which agrees with results seen in hepatocellular carcinoma in previous studies (Jiang et al., 2013). Next, we evaluated AKT and ERK signaling as known targets of celastrol (Pang et al., 2010; Tang et al., 2018). The result showed that high-dose celastrol (1 µM) reduced or sustained the levels of *p*-AKT and *p*-ERK in CRC cells at the 12th h, dependent on each cell line, whilst 0.25 µM celastrol strongly activated AKT and/or ERK signaling across three tested CRC cell lines (Figure 1B; Supplementary Figure S1). We then evaluated the contribution of AKT/ERK blockage (using corresponding inhibitors) in sensitizing celastrol. As shown in Figure 1C, either AKT or ERK inhibitor could significantly enhance the inhibitory effects of celastrol on CRC, especially those of low-dose celastrol (0.25–0.50 µM), which suggests a synergistic role. These results indicate that sustained AKT/ERK activation plays an important role in rendering low-dose celastrol ineffective against CRC.

### Receptor tyrosine kinase feedback weakens the inhibitory effect of low-dose celastrol on colorectal cancer cells

**FIGURE 2:**
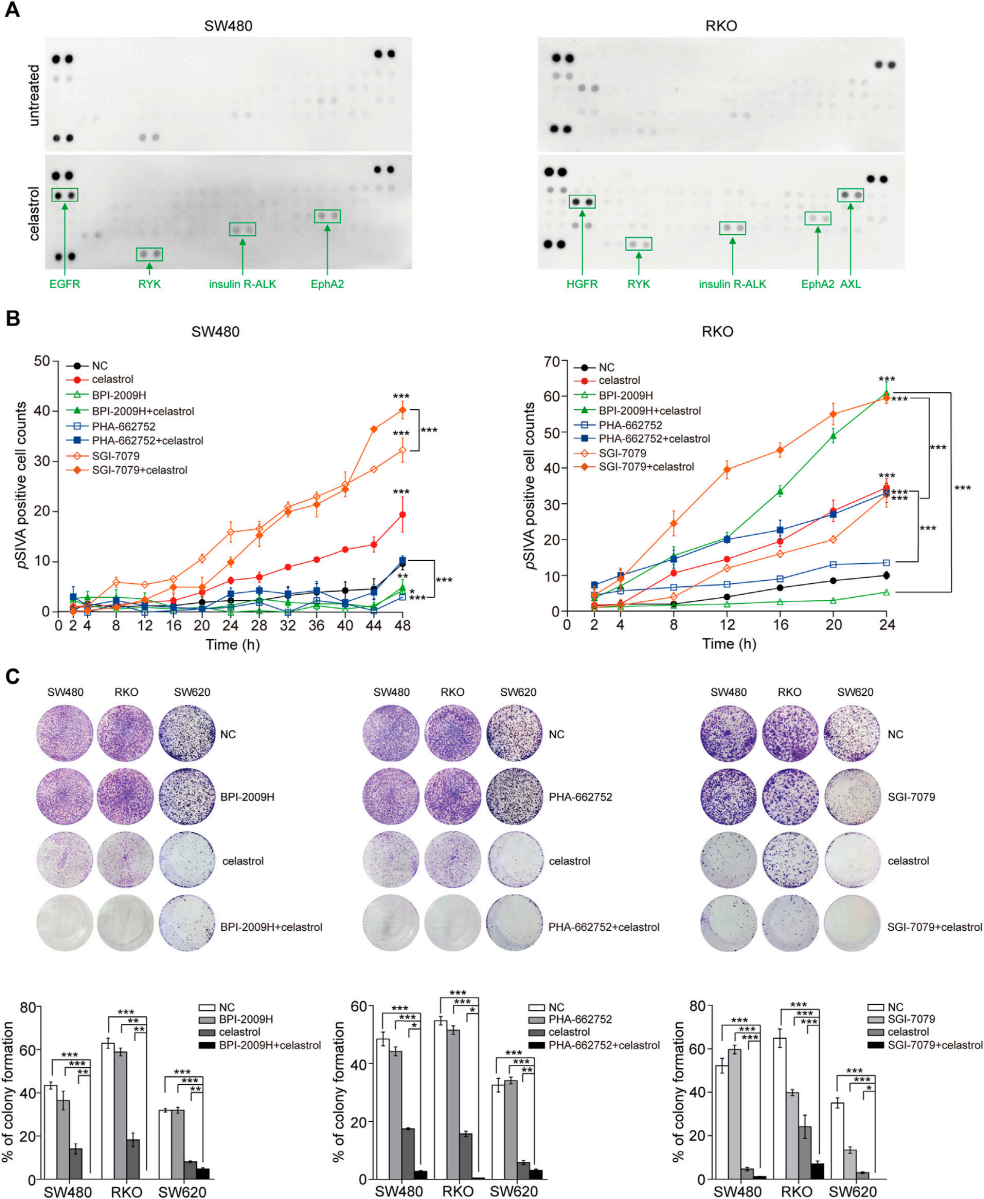
Reactivation of RTKs results in limited inhibitory effect of low-dose celastrol on CRC cells. **(A)** Activation of RTKs in CRC cells treated with 0.25 μM celastrol for 1 h. **(B)** *p*SIVA real-time apoptosis assay of CRC cells after the treatments of celastrol alone, RTK inhibitors alone, or their combination. **(C)** Colony formation assay of CRC cells received the treatment of celastrol alone, RTK inhibitor alone, or their combination. *p < 0.05; **p < 0.01; ***p < 0.001.

AKT/ERK activation usually associates with multiple RTKs. Therefore, we used RTK arrays to evaluate alterations of 49 RTKs in response to low-dose celastrol in three CRC cell lines. As expected, we observed that the levels of phosphorylated RTKs, including EGFR, EphA2, HGFR, AXL, insulin R, and ALK were increased in CRC cells after treatment with low-dose celastrol at the 12th h (Figure 2A; Supplementary Figure S2). However, the types and levels of RTKs affected were obviously inconsistent across the three CRC cell lines. To discover the contribution of different RTKs to celastrol-related CRC inhibition, we combined low-dose celastrol with the inhibitors corresponding to those elevated RTKs to test the cooperative effects. Dynamic apoptosis assays with *p*SIVA showed that the ALK inhibitor improved the proapoptotic role of low-dose celastrol on SW480 cells, while both AXL and EGFR inhibitors improved the proapoptotic role of celastrol on RKO cells (Figure 2B), indicating response heterogeneity to the combination of RTK inhibitors and celastrol. Using colony formation assays on three CRC cell lines, we observed that each inhibitor, corresponding to EGFR, HGFR, or ALK, enhanced the inhibitory effect of low-dose celastrol (Figure 2C), indicating that combination treatment is more effective for long-term inhibition of cell proliferation.

### The SHP2 inhibitor blocked the reactivation of receptor tyrosine kinase-derived AKT/ERK signaling by low-dose celastrol

**FIGURE 3:**
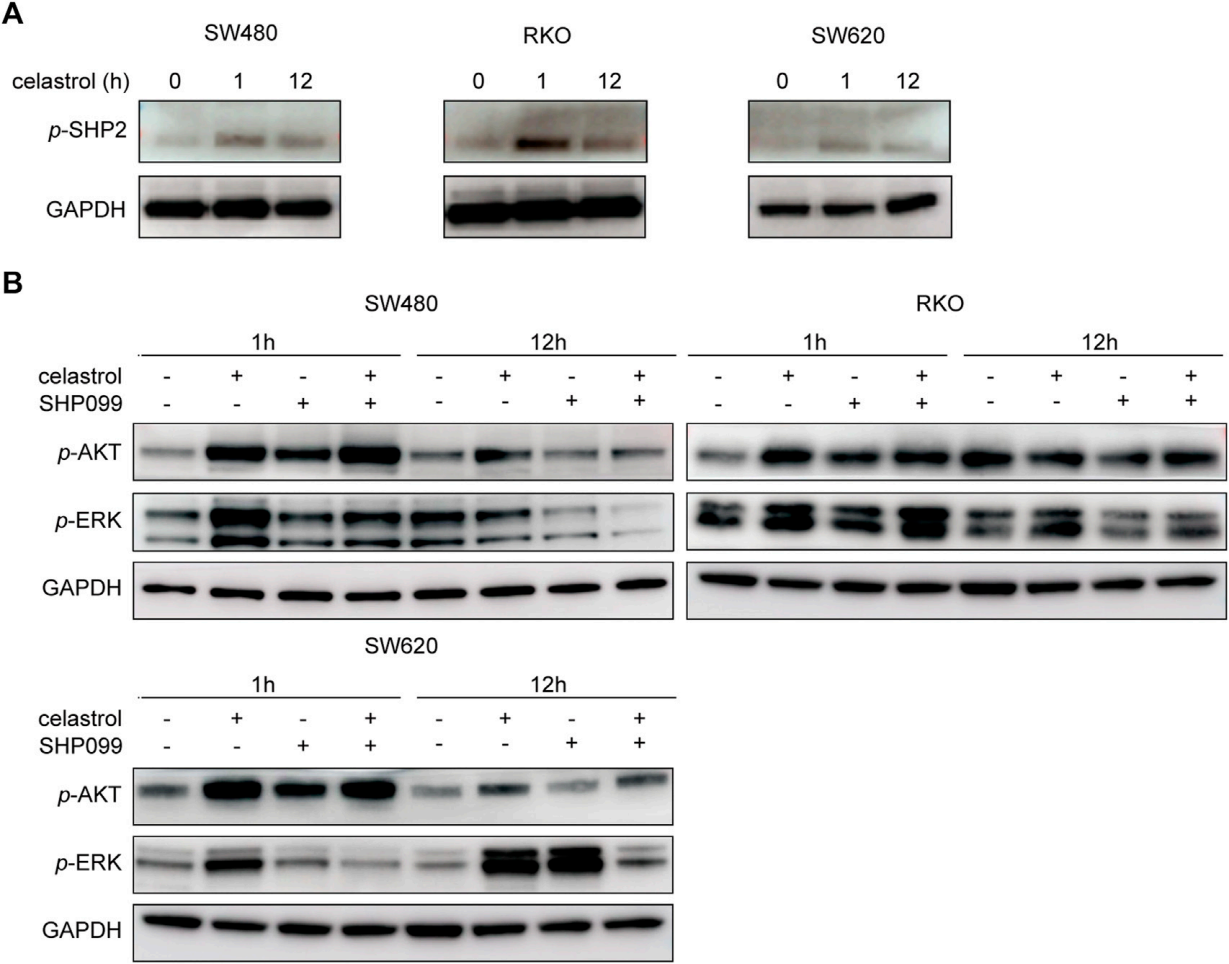
Inhibition of SHP2 blocked RTK-related AKT/ERK activation by low-dose celastrol. **(A)** Expression of *p*-SHP2 in CRC cells treated with 0.25 μM celastrol for 0, 1, and 12 h. **(B)** Expression of *p*-AKT and *p*-ERK in CRC cells treated with celastrol alone, SHP099 alone, or their combination for 1 and 12 h.

Considering that SHP2 is a well-known key mediator between multiple RTKs and AKT/ERK signaling, we next investigated the SHP2 status in response to low-dose celastrol and evaluated alterations in AKT/ERK signaling following the combination of SHP2 blockade and low-dose celastrol treatment. As shown in Figure 3A, consistent with the sustained increase of *p*-AKT and/or *p*-ERK (Figure 1B), *p*-SHP2 levels were obviously elevated in response to low-dose celastrol treatment in all three CRC cell lines, which suggests a critical role of SHP2 in the reactivation of AKT/ERK signaling by celastrol. Moreover, we observed that the combination of SHP099 and low-dose celastrol could remarkably suppress the increase of *p*-ERK and *p*-AKT, as compared with results observed with low-dose celastrol alone at 12 h (Figure 3B), which revealed the effectiveness of the combination of low-dose celastrol and SHP2 inhibitor.

### SHP2 blockage sensitized colorectal cancer cells to the inhibitory effect of low-dose celastrol

**FIGURE 4:**
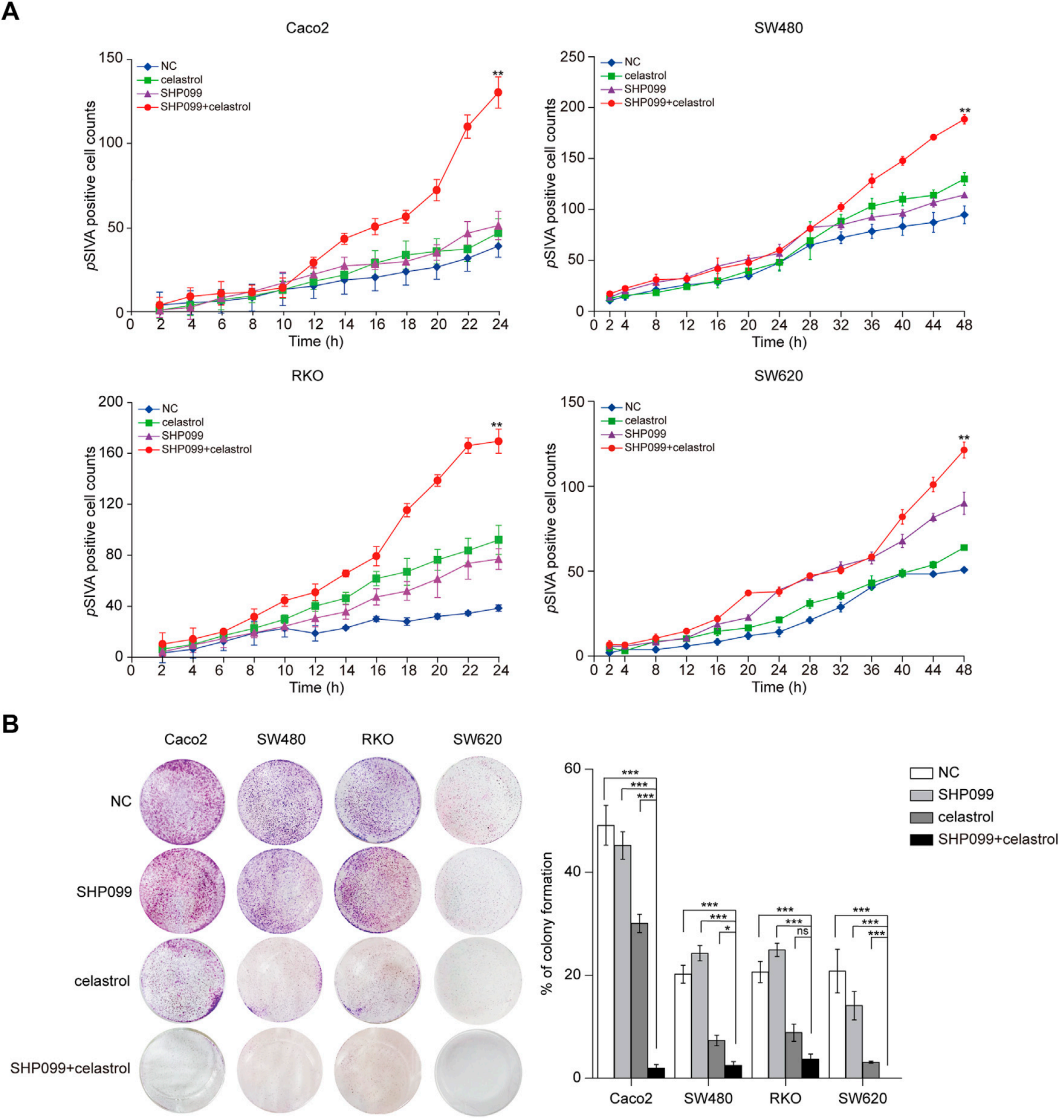
SHP099 cooperates with celastrol to enhance the apoptosis and growth inhibition of CRC cells. **(A)** *p*SIVA real-time apoptosis assay of CRC cells treated with celastrol and SHP099 with an indicated concentration. **(B)** Colony formation assay of CRC cells treated with celastrol alone, SHP099 alone, or their combination. ns, none sense; *p < 0.05; **p < 0.01; ***p < 0.001.

Next, the growth inhibitory effects of celastrol and SHP099 were evaluated using dynamic *p*SIVA staining assays and colony formation assays. The dynamic curves of *p*SIVA staining showed that the combination of low-dose celastrol and SHP099 reached the highest *p*SIVA staining ratio at the 24th h in Caco2/RKO cells and at the 48th h in SW480/SW620 cells, which revealed its strongest synergistic proapoptotic effect among all tested groups (Figure 4A; Supplementary Figure S3). Consistent with this result, this combination displayed outstanding effects compared to any single reagent (low-dose celastrol or SHP099 alone) across four CRC cell lines (Figure 4B).

### The SHP2 inhibitor and celastrol synergistically drove tumor regression in xenograft animal models

**FIGURE 5:**
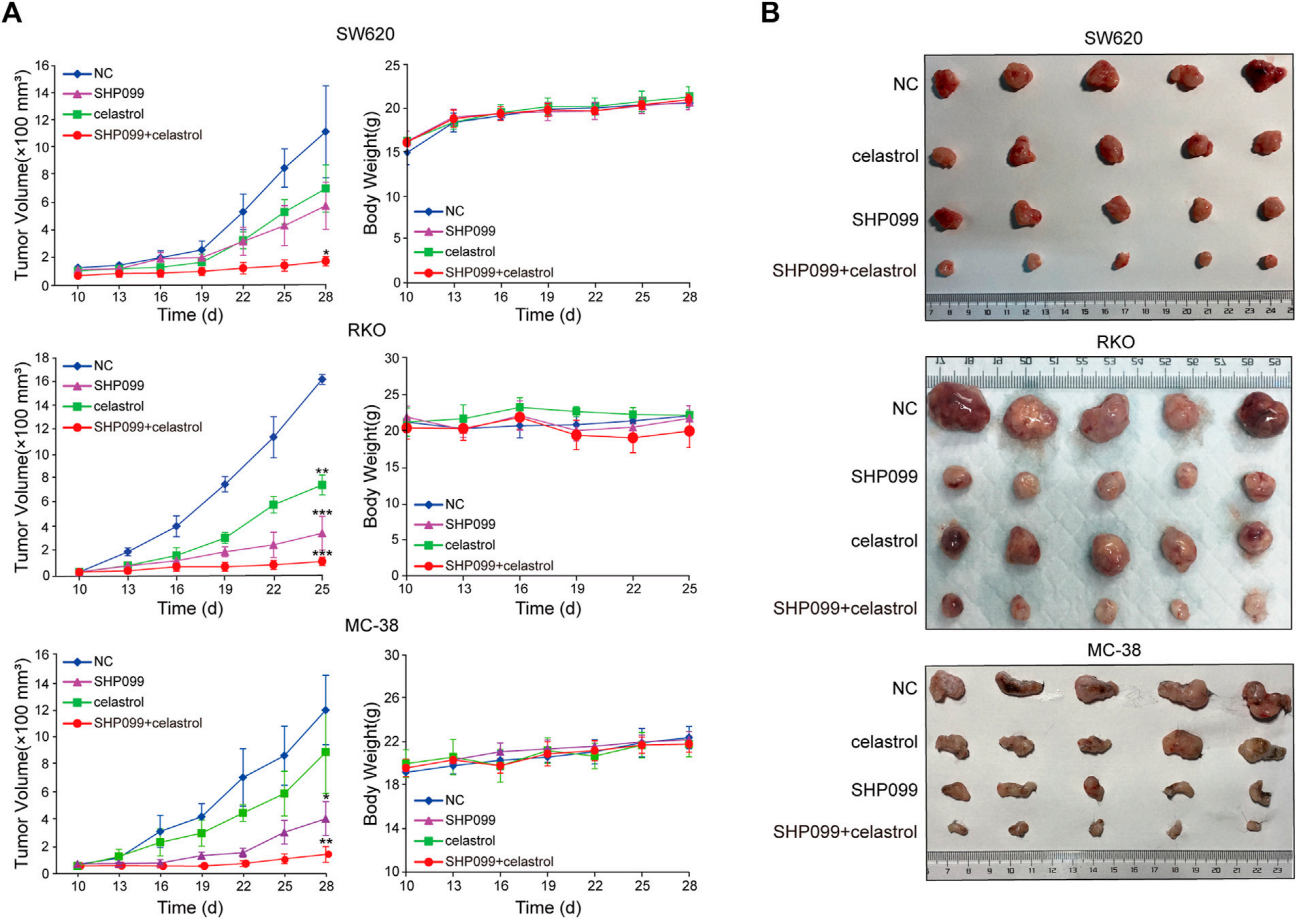
**S**HP099 cooperates with celastrol to suppress tumor growth in xenograft animal models. **(A)** Tumor volume and body weight variations after the treatment of celastrol alone, SHP099 alone, or their combination in animal models; **(B)**. representative images of tumors treated with celastrol alone, SHP099 alone, or their combination. *p < 0.05; **p < 0.01; ***p < 0.001.

Next, we set out to validate our *in vitro* findings using *in vivo* models. We injected SW620, RKO, and MC-38 cells into mice until the tumors reached the required volumes, approximately until the 10th day. Thereafter, daily oral dosing of SHP099, celastrol, or a combination of the two was employed according to the designed regimen. As shown in Figure 5A, tumor volumes exhibited significant differences among the four groups from 3–9 days after dosing. Although SHP099 alone and celastrol alone remarkably inhibited tumor growth, their combination yielded smallest tumor volumes among all CRC models. These tumors remained the closest in size to the initial sizes during the entire experimental period (Figure 5B). Moreover, compared with the reported body weight loss in mice treated with 2–4 mg/kg celastrol (Raja et al., 2011; Li et al., 2015), there was no significant difference in animal body weight among all groups in three CRC models, indicating that the regimen had not generated serious adverse effects on experimental animals (Figure 5A). The results from xenograft models supported the *in vitro* results indicating that a combination of SHP099 and low-dose celastrol may overcome adaptive feedback resistance and may present a promising therapeutic strategy.

### Immune activation by SHP2 inhibition confers the combined anti-colorectal cancer effect

**FIGURE 6:**
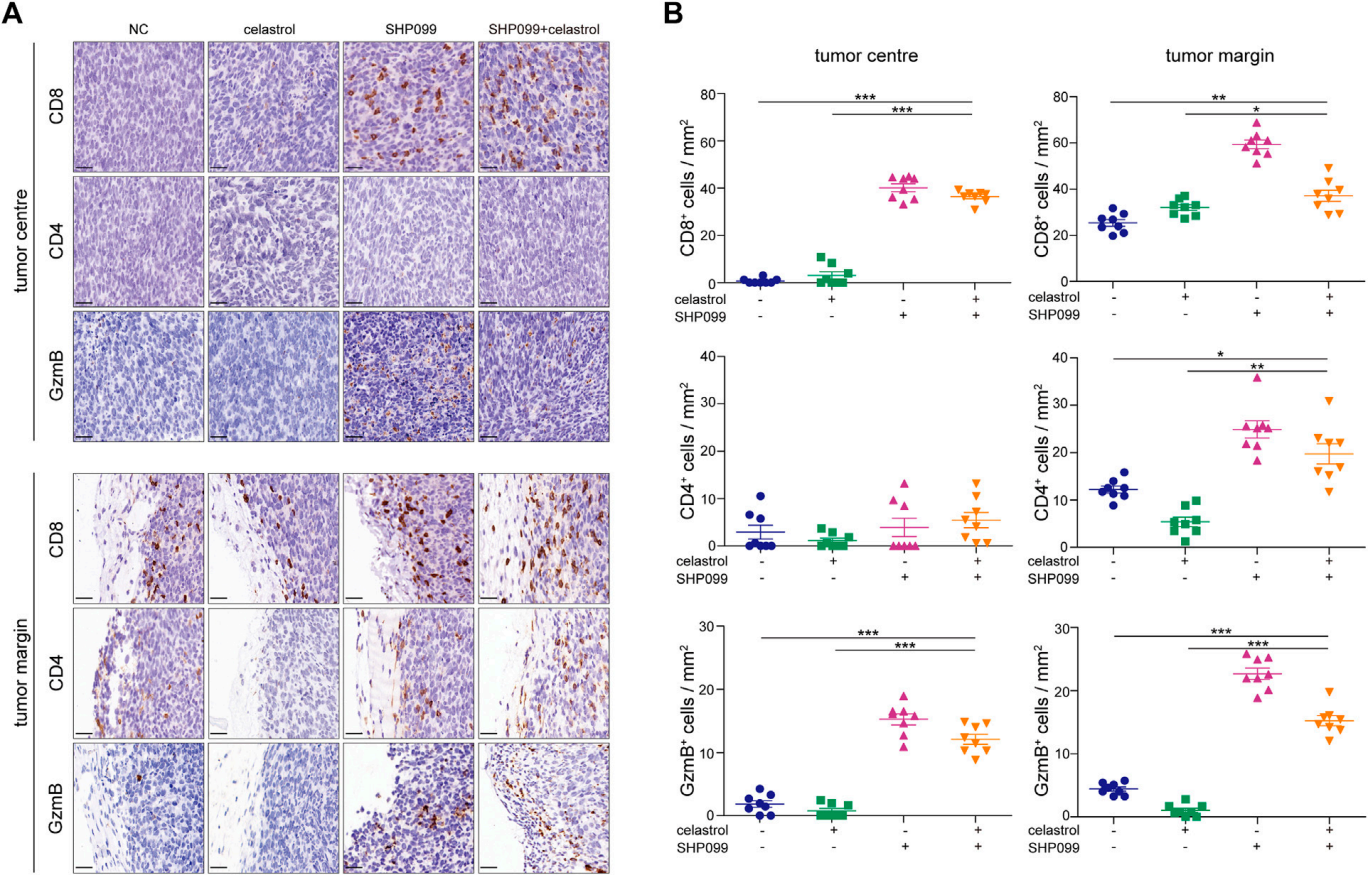
SHP099 enhances the antitumor role of celastrol by promoting T-cell infiltration. **(A)** Infiltration of CD8^+^, CD4^+^, and GzmB^+^ cells in CRC xenografts treated with SHP099 alone, celastrol alone, or their combination using IHC examination. **(B)** Quantification of CD8^+^, CD4^+^, and GzmB^+^ cells in tumors treated with SHP099 alone, celastrol alone, or their combination. *p < 0.05; **p < 0.01; ***p < 0.001. Scale bar: 20 μM.

Both celastrol and SHP2 inhibitors are involved in the regulation of T-cell function (Astry et al., 2015; Venkatesha et al., 2016), leading us to evaluate the effect of celastrol alone, SHP099 alone, and their combination on tumor immune infiltrates in MC-38 xenografts using IHC (Figure 6). As expected, we observed that SHP099 and the combination increased the density of infiltrated CD8^+^ cells in tumor and peritumoral areas as compared to the control subgroup, while celastrol treatment only increased the infiltrates of CD8^+^ cells in peritumoral areas. No significant difference was observed in the density of CD4^+^ cells inside the tumor among four subgroups. However, in peritumoral areas, celastrol treatment decreased the density of CD4^+^ cells, while SHP099 and the combined treatment strongly promoted the invasion of CD4^+^ cells. Furthermore, we observed that celastrol slightly reduced the infiltration of GzmB^+^ cells, while SHP099 treatment showed the opposite effect on both tumor and peritumoral areas, and the combination treatment produced a result in between singular treatments. These results indicated that celastrol and SHP2 inhibition may have opposing roles in T-cell function, while their combination may greatly improve the immune-suppression drawback of celastrol.

## Discussion

High-dose celastrol exhibits potential anticancer activity. However, the severe side effects greatly limit its clinical application. Therefore, combination therapy with low-dose celastrol is a promising strategy to overcome the compensatory mechanisms and to reduce unwanted off-target effects. In this study, we found that AKT and ERK signals are constantly activated in response to low-dose celastrol compared to high-dose celastrol, which transiently activates the signals. Since failure to inhibit AKT and ERK signals are usually associated with drug resistance, we hypothesized that blocking these signals may enhance the sensitivity of CRC cells to low-dose celastrol. As expected, low-dose celastrol generated synergistic inhibitory effects on CRC cells when combined with the inhibitor of AKT or ERK in our CRC proliferation assays. In addition to AKT and ERK signaling, many targets of celastrol have been reported, such as HSP90 and STAT3, which are all important for the aggressiveness of CRC cells (Zhu et al., 2020). Our data demonstrated that the combination of low-dose celastrol with the inhibitors, which only block some known targets of celastrol, produces obvious inhibitory effects on CRC growth compared to each drug alone. These effects may possibly occur simultaneously and with lower toxicity than high-dose celastrol.

AKT and ERK signal reactivation in response to low-dose celastrol may be complex, with the most common mechanism involving RTK signaling feedback. Previous studies have reported that the inhibitors corresponding to several RTKs, such as EGFR (Dai et al., 2021), ERBB2 (Raja et al., 2011; Chen et al., 2020) and FGFR, can generate synergistic effects on cancer cell growth in combination with celastrol, supporting the idea that the reactivation of AKT/ERK signals by celastrol may be caused by RTK feedback. Using phosphorylated RTK arrays, we confirmed that several RTKs are activated by low-dose celastrol, including EGFR, HGFR, and AXL, but the RTK profiles displayed obvious heterogeneity across different cell lines. Furthermore, the combination of low-dose celastrol and the inhibitor against EGFR, HGFR, or AXL resulted in stronger inhibition of CRC cell growth than each inhibitor alone, demonstrating that blocking reactive RTK signals may enhance the efficacy of low-dose celastrol against CRC cells.

**FIGURE 7:**
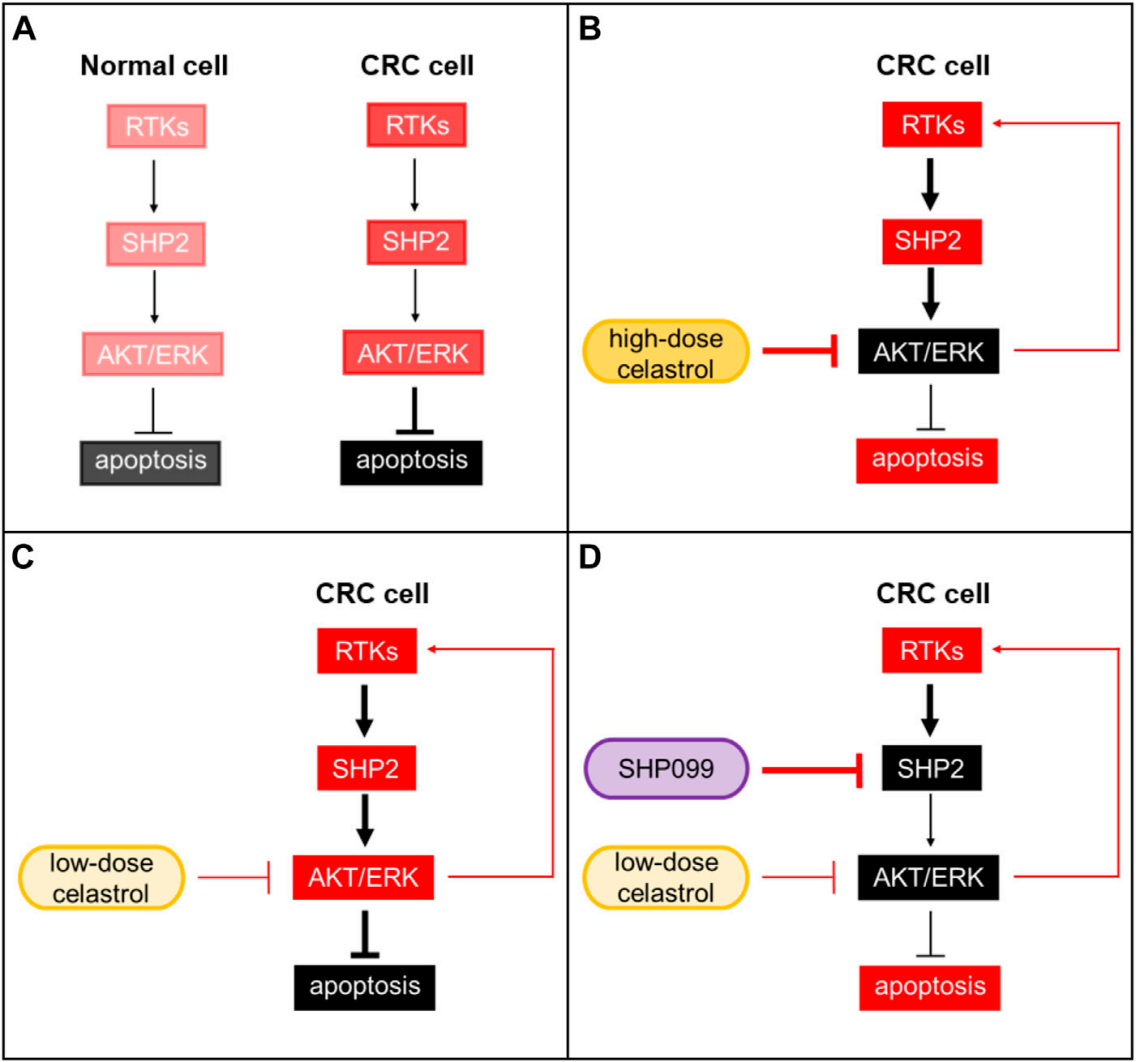
SHP2 inhibition improves the tumor growth suppression of celastrol in CRC. **(A)** Background activation of the RTKs-SHP2-AKT/ERK pathway in normal and CRC cells. **(B)** CRC under high-dose celastrol treatment. Activation of AKT/ERK was strongly suppressed. **(C)** CRC under low-dose celastrol treatment. Feedback reactivation of AKT/ERK through the RTKs-SHP2-ARK/ERK axis showed a dominant effect. **(D)** CRC under combined treatment of low-dose celastrol and SHP099. SHP099 and low-dose celastrol cooperatively reduced *p*-AKR/*p*-ERK and resulted in a synergic anti-CRC effect. Red: activated; black: suppressed.

Although success was observed with celastrol and RTK inhibitor combination, the efficacy of combination therapy may depend on whether celastrol-induced RTKs are inhibited. SHP2 is known as a key mediator of multiple RTKs, and its inhibitors have been reported in recent years. Inhibition of SHP2 combined with inhibitors of KRAS^G12C^, RAF, or MEK has been shown to generate deep growth suppression in multiple preclinical cancer models, and these underlying mechanisms involve RTK feedback blockade by SHP2 inhibitors. We found that levels of phosphorylated SHP2 were consistently increased across several CRC cell lines by low-dose celastrol alone, and the combination of SHP099 (a selective SHP2 inhibitor) with low-dose celastrol obviously suppressed CRC cell growth in both *in vitro* and *in vivo* models. In cells under high-dose celastrol treatment, the feedback reactivation of the RTKs-SHP2-ARK/ERK axis was also suppressed, but in cells under low-dose celastrol treatment, the feedback pathway showed a dominant effect. Additional inhibition of SHP2 would greatly block this feedback pathway so that presented synergic anti-CRC effect with low-dose celastrol (Figure 7). This evidence shows that a treatment combination of celastrol and SHP2 inhibitor may be a promising regimen.

In addition to blocking RTK-AKT/ERK signal feedback in response to celastrol via SHP2 inhibition, the combination of SHP099 and celastrol may also regulate the tumor immune microenvironment. Celastrol reportedly directly suppresses T-cell proliferation and Th17 cell induction, while facilitating forkhead box P3 (Foxp3) expression and Treg cell proliferation, indicating an immune suppression phenotype in cancer (Astry et al., 2015; Zhang et al., 2018). However, we observed that infiltration of CD8^+^ cells in tumors that received celastrol was slightly higher than that in control tumors, while CD4^+^ cell infiltration exhibited opposite results. It was recently reported that celastrol can also enhance antitumor immune activation by inducing immunogenic cell death (ICD) and reducing the expression of PD-L1 (Qiu et al., 2021), or by upregulating tumor death receptors and then enhancing T-cell cytotoxicity (Li et al., 2016). SHP2 is also widely expressed in hematopoietic cells, including both lymphoid and myeloid cells, and there is emerging evidence to support its role in tumor immunity (Liu et al., 2017; Liu et al., 2020; Quintana et al., 2020; Wang et al., 2021). Research has documented a negative role of SHP2 in T-cell activation. In CD4^+^ T lymphocytes, two major phosphatase targets of SHP2 are STAT1 (which triggers INF secretion) and STAT3 (which promotes IL-17A production). Dephosphorylation of STAT1 and STAT3 by SHP2 results in their inactivation and inhibition of their downstream signal transduction. Given the possibility that SHP2 suppression would contrariously facilitate antitumor immunity. As expected, we observed increased infiltration of CD8^+^, CD4^+^, and GZMB^+^ cells in the margin and/or middle region of tumors exposed to SHP099. Furthermore, anti-tumor immune cell infiltration was greater after combined treatment with SHP099 and celastrol than that observed in the celastrol group, but lower than that in the SHP099 group, suggesting that the combination of SHP099 would overcome the immune suppression of celastrol and in this way SHP099 further supported the tumor growth suppression of celastrol. The mechanism of T-cell activation in this system is complicated and remained a future study.

Collectively, we found that blocking RTK-ERK/AKT signaling enhances the inhibitory efficacy of low-dose celastrol on CRC growth. The combination of celastrol and SHP099 remarkably suppresses CRC growth in both *in vitro* and *in vivo* models. The underlying mechanism for the combination regimen may involve RTK feedback blockade and enhanced antitumor immune activation via SHP2 inhibition even during celastrol treatment. Although elevated anti-CRC function was observed, considering the fact that CRC patients hold complicated mutational loads, such as diverse KRAS or BRAF mutations, our combination regimen still needs further confirmation in various CRC cells to be applied to clinical use.

## Data Availability

The original contributions presented in the study are included in the article/Supplementary Material; further inquiries can be directed to the corresponding authors.
