# Supplementary material for: SHP2 inhibition improves celastrol-induced growth suppression of colorectal cancer
Source: Front Pharmacol. 2022 Sep 1;13:929087. doi: 10.3389/fphar.2022.929087 (PMC9477229; doi:10.3389/fphar.2022.929087)
Supplement: Supplementary file 1 [file DataSheet2.PDF]

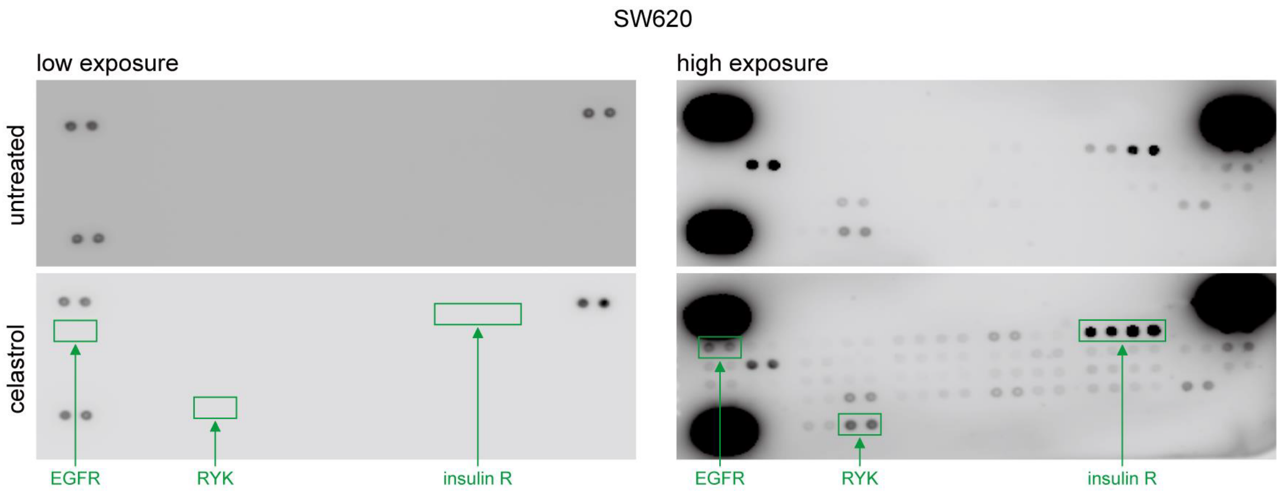

Supplementary Figure 2 . Activation of RTKs in SW620 treated with 0.25 μM celastrol for 12 h. Left panel: Images under low exposure; Right panel: Images under high exposure.
