# Supplementary material for: SHP2 inhibition improves celastrol-induced growth suppression of colorectal cancer
Source: Front Pharmacol. 2022 Sep 1;13:929087. doi: 10.3389/fphar.2022.929087 (PMC9477229; doi:10.3389/fphar.2022.929087)
Supplement: Supplementary file 2 [file DataSheet3.PDF]

A

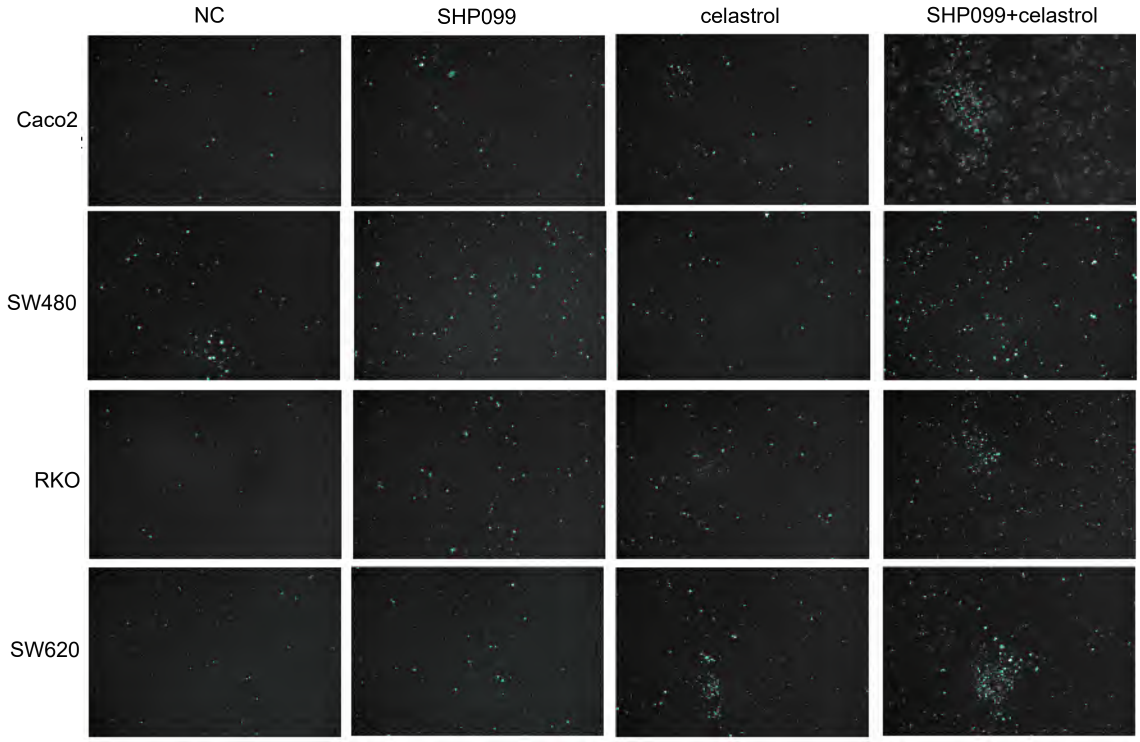

Supplementary Figure 3. Images of *pSIVA* real-time apoptosis assay of CRC cells treated with celastrol (0.25 μM), SHP099 (20 μM), and their combination in 24 h (Caco2 and RKO) or 48 h (SW480 and SW620). GFP^+ dots were calculated for quantification.
