# Supplementary material for: SHP2 inhibition improves celastrol-induced growth suppression of colorectal cancer
Source: Front Pharmacol. 2022 Sep 1;13:929087. doi: 10.3389/fphar.2022.929087 (PMC9477229; doi:10.3389/fphar.2022.929087)
Supplement: Supplementary file 3 [file DataSheet1.PDF]

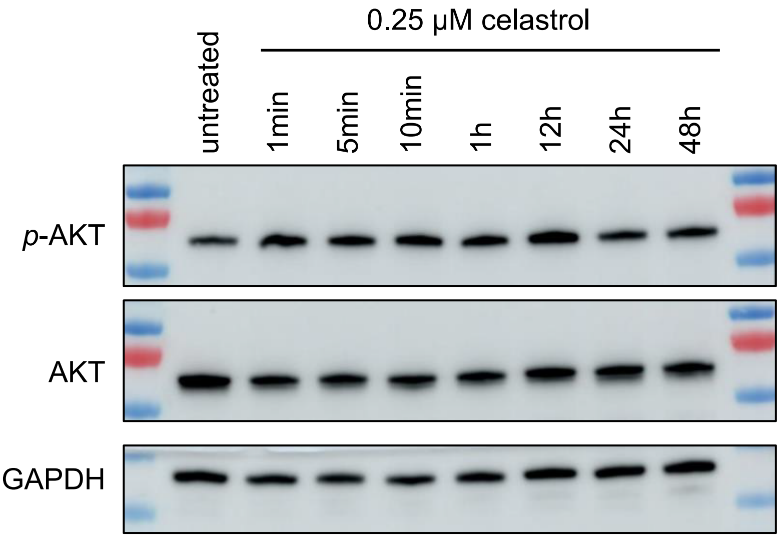

Supplementary Figure 1. Low-dose celastrol treatment resulted in sustained AKT activation in CRC cells. RKO cells were treated with 0.25 μM celastrol for 1 min to 48 h. Cell lysate was collected and expression of *p*-AKT (CST, 4060), AKT (CST, 4691) and GAPDH (abcam, ab181602) was detected by western blot.
